# COMplex Fracture Orthopedic Rehabilitation (COMFORT) - Real-time visual biofeedback on weight bearing versus standard training methods in the treatment of proximal femur fractures in the elderly: study protocol for a multicenter randomized controlled trial

**DOI:** 10.1186/s13063-018-2612-9

**Published:** 2018-04-12

**Authors:** Marco Raaben, Syaiful Redzwan, Robin Augustine, Taco Johan Blokhuis

**Affiliations:** 1 Department of Surgery, University Medical Center Utrecht, Heidelberglaan 100, 3508 GA Utrecht, The Netherlands; 2 Department of Engineering Sciences, Uppsala University, Lägerhyddsvägen 1, SE751 21 Uppsala, Sweden; 3 Department of Surgery, Maastricht University Medical Center+, P. Debyelaan 25, 6229 HX Maastricht, The Netherlands

**Keywords:** Proximal femur fracture, Weight-bearing, Biofeedback, Gait analysis, SensiStep, Fracture rehabilitation

## Abstract

**Background:**

Proximal femur fractures are a common injury after low energy trauma in the elderly. Most rehabilitation programs are based on restoring mobility and early resumption of weight-bearing. However, therapy compliance is low in patients following lower extremity fractures. Moreover, little is known about the relevance of gait parameters and how to steer the rehabilitation after proximal femur fractures in the elderly. Therefore, the aim of this prospective, randomized controlled trial is to gain insight in gait parameters and evaluate if real-time visual biofeedback can improve therapy compliance after proximal femur fractures in the elderly.

**Methods:**

This is a two-arm, parallel-design, prospective, randomized controlled trial. Inclusion criteria are age ≥ 60 years, a proximal femur fracture following low energy trauma, and unrestricted-weight bearing. Exclusion criteria are cognitive impairment and limited mobility before trauma. Participants are randomized into either the control group, which receives care as usual, or the intervention group, which receives real-time visual biofeedback about weight-bearing during gait in addition to care as usual. Spatiotemporal gait parameters will be measured in 94 participants per group during a 30-m walk with an ambulatory biofeedback system (SensiStep). The progress of rehabilitation will be evaluated by the primary outcome parameters maximum peak load and step duration in relation to the discharge date. Secondary outcome parameters include other spatiotemporal gait parameters in relation to discharge date. Furthermore, the gait parameters will be related to three validated clinical tests: Elderly Mobility Scale; Functional Ambulation Categories; and Visual Analogue Scale. The primary hypothesis is that participants in the intervention group will show improved and faster rehabilitation compared to the control group.

**Discussion:**

The first aim of this multicenter trial is to investigate the normal gait patterns after proximal femur fractures in the elderly. The use of biofeedback systems during rehabilitation after proximal femur fractures in the elderly is promising; therefore, the second aim is to investigate the effect of real-time visual biofeedback on gait after proximal femur fractures in the elderly. This could lead to improved outcome. In addition, analysis of the population may indicate characteristics of subgroups that benefit from feedback, making a differentiated approach in rehabilitation strategy possible.

**Trial registration:**

TrialRegister.nl, NTR6794. Registered on 31 October 2017.

**Electronic supplementary material:**

The online version of this article (10.1186/s13063-018-2612-9) contains supplementary material, which is available to authorized users.

## Background

Due to the ageing population, the worldwide incidence of hip fractures will rise from 1.66 million in 1990 to 6.26 million by 2050 [1]. Hip fractures have a high mortality and high morbidity [2]. Loss of function is common after a hip fracture and patients experience difficulties in their return to society or to their previous habitat. This leads to long-term care in rehabilitation facilities, which is the largest component in the total costs in the treatment of hip fracture patients [3].

Most rehabilitation programs are based on restoration of mobility and early resumption of weight-bearing [4]. Weight-bearing is important as it helps to maintain muscle and bone mass [5]. In addition, early weight-bearing could also lead to improved fracture healing through mechanotransduction [6]. Therefore, early (partial) weight-bearing is generally advocated and trained with walking aids under supervision of a physical therapist. Bathroom scales are commonly used to guide the amount of weight-bearing to the patient. This method is not only unreliable, but also not very helpful for patients as information about the amount of weight-bearing is only provided in the static situation and lost in the dynamic situation.

Although the bathroom scale is still the gold standard in most rehabilitation clinics, the introduction of biofeedback systems is promising as information can be provided in the dynamic situation [7]. Previous studies have already shown improvement in partial and full weight-bearing when biofeedback systems were used, compared to standard training methods [8, 9]. For example, visual biofeedback has shown positive results in the treatment after Parkinsons’s disease, in the late period after stroke, and after cerebral palsy [10–12]. Also, auditory and visual biofeedback have shown significant improvements in weight-bearing after lower extremity fractures [9, 13, 14]. Although these results seem promising, the effect of real-time visual biofeedback on weight-bearing during rehabilitation after proximal femur fractures in the elderly is still unknown.

Therefore, in this randomized controlled trial (RCT), real-time visual biofeedback will be provided to the patient and healthcare professional in the clinical setting by the ambulatory biofeedback system SensiStep (Evalan BV, Amsterdam, The Netherlands). This biofeedback system was previously validated in static and dynamic situations [15]. In short, the system consists of an in-sole force sensor, which is able to measure weight-bearing reliably and continuously during gait. Generated peak loads are directly translated into a LED signal, to provide real-time feedback to the patient. The same signal is sent to a tablet, resulting in a real-time graphical illustration of each step shown to the healthcare professional. By using this biofeedback system, patients can be guided to the optimal level of weight-bearing. This potentially leads to better functional outcome, as well as improved fracture healing through mechanotransduction.

In this RCT, it will be investigated whether elderly patients recover faster or better after proximal femur fractures if they receive real-time visual biofeedback in the clinical setting. The control group receives care as usual and the intervention group receives care as usual with the addition of real-time visual biofeedback to improve weight-bearing to the optimal level. Hypothetically, patients in the intervention group show: (1) an increase in maximum peak loads; and (2) faster step durations at an earlier timepoint in their rehabilitation. Furthermore, patients in the intervention group recover faster, which results in an earlier discharge date.

## Methods

### Study design

This protocol is described according to the SPIRIT (Standard Protocol Items: Recommendations for Interventional Trials) Statement (see Additional file 1). The study is an international, multicenter trial conducted between March 2017 and August 2018 in the Netherlands and Sweden. Rehabilitation centers focused on geriatric rehabilitation are asked to participate in this study. In order to ensure the quality and expertise of the participating centers, a minimum inclusion rate of at least 20 patients per year is expected. Therefore, the number of eligible patients is screened in each center before their participation in the study. Measurements will be performed by trained physical therapists at the site and all physical therapists are experienced in geriatric trauma care. Before the start of the study, all physical therapists agreed on the study treatment regime during multiple consensus meetings and were trained to uniformly implement this study treatment. Participating geriatric rehabilitation clinics are listed in Table 1.

**Table 1:** Participating rehabilitation clinics

Clinic	Place	Allocation (group)
Zorgspectrum *Geinsche Hof* *Vuurscheschans 75* *3432 TX Nieuwegein*	Nieuwegein (NL)	Intervention
Warande *Bovenwegen* *Heideweg 2* *3708 AT Zeist*	Zeist (NL)	Intervention
Warande *Diakonessenhuis* *Professor Lorentzlaan 76* *3707 HL Zeist*	Zeist (NL)	Intervention
Beweging 3.0 *Meander Medical Center* *Maatweg 3* *3813 TZ Amersfoort*	Amersfoort (NL)	Control
Evean *Schoenerstraat 11* *1034 XZ Amsterdam*	Amsterdam / Zaandam (NL)	Intervention
Beweging 3.0 *Woonzorgcentrum De Pol* *Vetkamp 85* *3862 JN Nijkerk*	Nijkerk (NL)	Control
Cordaan *In het Zomerpark* *Remmersteinpark 3–5* *2151 KE Nieuw-Vennep*	Nieuw-Vennep (NL)	Control
azM Herstelzorg *Sint Pieterstraat 23* *6211 JM Maastricht*	Maastricht (NL)	Control
Zorggroep Groningen *Schaaksport 100–102* *9728 PG Groningen*	Groningen (NL)	Intervention
Careyn *Nieuw Tamarinde* *Neckardreef 6* *3562 CN Utrecht*	Utrecht (NL)	Control
Telge Rehab *Östra Kanalgatan 2* *152 71 Södertälje*	Södertälje (SE)	Control

Participating geriatric rehabilitation clinics in The Netherlands and Sweden. Clinics were allocated to either the control or the intervention group

This trial is a two-arm, parallel-design, prospective RCT. Based on a previous pilot study, the average length of rehabilitation is 44 days, with a standard deviation of 17. Using a power of 0.80, an alpha of 5%, and an expected reduction of seven days (one week) in two-sided testing, a sample size of 94 participants per group was calculated. Participants will be allocated to either the control group or the intervention group using a stratified cluster randomization according to the sealed opaque envelope principle. First, all rehabilitation clinics were divided into two groups depending on the inclusion rate: < 2 vs. ≥ 2 eligible participants per month. This division was made to avoid skewing of inclusion rate in both arms. Then in both groups, clinics were randomized by the principal investigator into the control or intervention group using the sealed opaque envelope principle. This resulted in six rehabilitation clinics in the control group and five rehabilitation clinics in the intervention group (Table 1). Randomization per center, or cluster randomization, was chosen for two reasons. First, individual selection bias by the physical therapist is minimized. Second, and more importantly, the physical therapists and participants within one center can now provide a single treatment, either care as usual or care as usual, with real-time visual biofeedback. Considering the constant interaction between physical therapists, as well as between participants, individual randomization would increase the risk of data collection errors.

### Participants

Strict inclusion criteria will be used to introduce homogeneity of the groups; therefore, participants enrolled in the study meet the following inclusion criteria:

1. The participants rehabilitate from a proximal femur fracture following low energy trauma (e.g. fall from standing position).
2. The participants have a prescribed unrestricted weight-bearing after (surgical) treatment of their fracture.
3. The participants have an expected clinical rehabilitation duration of ≥ 2 weeks.
4. The participants are aged ≥ 60 years.
5. The participants have a bodyweight ≤ 120 kg.

Participants with the following criteria will be excluded from the study:

1. People with cognitive impairment, defined as a score < 18 on the Mini Mental State Examination (MMSE) [16].
2. People who are readmitted to the hospital within two weeks after study participation, for example for infectious complications.
3. People with co-morbidities that affected gait significantly before the proximal femur fracture.

### Protocol

**Fig. 1:**
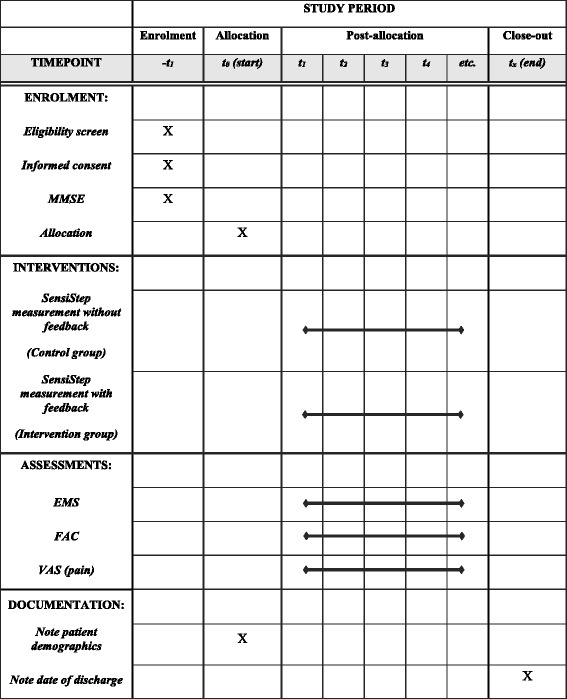
Schedule of enrolment, interventions, assessments, and documentation. MMSE Mini Mental State Examination, EMS Elderly Mobility Scale, FAC Functional Ambulation Categories, VAS Visual Analogue Scale

Data will be gathered according to a strict study protocol (Fig. 1). Candidate patients who fulfill the inclusion criteria will be asked to participate in the study by the site investigator and receive an information letter with the study protocol and study objectives. If patients agree to participate in the study, informed consent will be signed and the cognitive score will be evaluated by the MMSE. The MMSE is an objective score to quantitatively asses the severity of cognitive impairment. A MMSE score < 18 indicates severe cognitive impairment [16] and this leads to exclusion of the patient in this trial. After informed consent and MMSE, data will be collected from participants according to the following trial protocol. The institutional protocol for physical therapy after proximal femur fractures will be followed in all participants. In addition, each participant receives daily force measurements using the SensiStep system in a 30-m walk to measure the gait parameters. Additional clinical tests will be executed to gain additional insight in the rehabilitation progress of each participant:

1. Functional Ambulation Categories (FAC, once per week)
2. Elderly Mobility Scale (EMS, twice per week)
3. Visual Analogue Scale (VAS, daily)

Finally, additional information of each participant will be documented, including the following:

1. Date of surgery
2. Type of surgery
3. Co-morbidities
4. Walking aid
5. Length of admission to the rehabilitation center
6. Age
7. Gender
8. Length
9. Weight

### Interventions

Participants in the intervention group receive, in addition to the standard institutional protocol, real-time visual feedback about weight-bearing during the 30-m walk with SensiStep. The physical therapist has real-time visual insight in the weight-bearing as well by observing the tablet together with the participant. Both the biofeedback system (visually) and the physical therapist (verbally) assist and motivate the participant to adapt the weight-bearing to the optimal level. The target weight will be set at 100% bodyweight, as the participants have no restrictions in weight-bearing.

### Outcome measures

The primary outcome measures are the gait parameters maximum peak load (in %bodyweight) and step duration (in seconds), which will be analyzed in relation to the discharge date from the rehabilitation center. It is expected that the intervention group will show an improvement in gait parameters and earlier discharge date compared to the control group. The discharge date will be determined by a multidisciplinary team within the usual institutional regime, irrespective of the study protocol. Secondary outcomes include other spatiotemporal gait parameters and validated clinical tests, including the EMS, the FAC, and the VAS. The EMS is a standardized validated scale for assessment of frail elderly people. The FAC is used as a diagnostic and evaluative tool to assess the physical performance of patients. The VAS is a commonly used, subjective scale to assess the amount of pain patients experience. Spatiotemporal gait parameters measured by the SensiStep will be correlated to the clinical test scores (e.g. EMS, FAC, and VAS). Blinding the participants and/or physical therapist is not possible, as both users are aware of the biofeedback they receive in the intervention group. However, data will be encrypted and anonymously delivered by the Steering Committee to the data scientists, which makes the data scientists blinded for the analysis of gathered data.

### Statistical analysis

Statistical analysis on outcome measures will be conducted using Statistical Package for the Social Sciences (SPSS). First, data distribution will be assessed. Second, missing data will be handled by a multiple imputation technique. The primary analysis will be based on an intention-to-treat principle. Also, a per-protocol analysis will be performed as sensitivity analysis to assess the robustness of the results to protocol deviations [17]. The primary goal is to find a relation between the gait parameters (maximum peak load and step duration) and the discharge date. In addition, other spatiotemporal gait parameters measured by the SensiStep will be analyzed for this relation as well. The secondary goal is to find a relation between the gait parameters and the clinical measurements (i.e. EMS, FAC, and VAS). Relations between all data will be examined using multiple regression analysis.

### Monitoring

This trial will be conducted according to the principles of the Declaration of Helsinki (amended version by the 64th WMA General Assembly, Fortaleza, Brazil, October 2013) and in accordance with the Medical Research Involving Human Subjects Act. Gathered data will be encrypted and stored on a secure server and is accessible by the Steering Committee.

This trial will be conducted under strict supervision of an experienced Steering Committee from the University Medical Center Utrecht (UMCU), Maastricht University Medical Center+ (MUMC+), and Uppsala University (UU). Acting as a Steering Committee, the principal investigators from UMCU and MUMC+ will discuss the progress of enrollment at least once every two weeks. The Steering Committee will perform a blinded interim analysis on 30%, 60%, and 90% of the included participants. During these interim analysis, reasons for exclusion will be discussed with the site investigators. Other items will be discussed as well, including but not limited to, applied discharge criteria for each patient, adverse events, quality of the retrieved data, missing data, and necessity to include other participating centers.

A monthly newsletter will be written by the Steering Committee and distributed among the participating centers. In this newsletter, the inclusion rate will be shown per center, which is a common and effective way to increase awareness and readiness to include patients in multicenter trials. The trial progress and general results will be communicated by the Steering Committee upon request.

## Discussion

The worldwide incidence of hip fractures among the elderly will increase due to the ageing population [1]. As hip fractures have a high morbidity and mortality, improvements should be made in the treatment of hip fractures. Biofeedback system have previously shown their potential, as weight-bearing significantly improved in partial and full weight-bearing [9]. This could improve fracture healing through mechanotransduction [6]. As stated before, the hypothesis is that real-time visual biofeedback results in a significant improvement in weight-bearing in the elderly after proximal femur fractures, especially with respect to the gait parameters maximum peak load and step duration, compared to standard training methods. Second, it is hypothesized that improvements in spatiotemporal gait parameters will lead to improvements in clinical scores, such as the EMS, FAC, and VAS. The aim of our trial is to validate these hypotheses and investigate the effect of real-time visual biofeedback on weight-bearing during the rehabilitation after proximal femur fractures in the elderly. This potentially leads to improved rehabilitation (e.g. better functional outcome), faster rehabilitation, and lower medical costs.

Although extensive attention has been paid to the study design, some difficulties still remain. One of the difficulties in this trial is the study population. Often, the elderly who sustain a hip fracture due to low energy trauma have (multiple) co-morbidities that could negatively affect gait. It is difficult to detect the amount of influence of these co-morbidities on gait. The question arises if any improvements or deterioration in gait is caused by real-time visual biofeedback or by the co-morbidities. To overcome this issue, the strict inclusion criteria for participants should minimize variability in the data. Moreover, the large number of participants (e.g. 94 per arm) should minimize the effect of co-morbidities on gait.

The topic of adherence could be another issue in this trial, as the trial will cause additional burden to both the participants and physical therapists. It is therefore important to select enthusiastic and experienced rehabilitation centers as candidate centers. This will be determined by asking candidate centers to register all eligible patients before the start of this trial over two months. Furthermore, the Steering Committee will weekly evaluate the inclusion rate and search for missing data, which provides the opportunity to steer directly if any issues are detected.

The potential effect of real-time visual biofeedback on weight-bearing can have a major influence on rehabilitation after proximal femur fractures in the elderly; therefore, dissemination is important. A stakeholder analysis will be performed early in the project and will determine how dissemination to these stakeholders should be handled in order to convince them of the added value of real-time visual biofeedback. The stakeholder analysis will identify which organizations and people the dissemination should target. Dissemination will take place at different levels, most importantly the patient level, the care provider level, and the level of the healthcare organizations, including insurance companies.

In summary, this is a multicenter RCT to investigate the rehabilitation progress and the value of real-time visual biofeedback on weight-bearing during rehabilitation after proximal femur fractures in the elderly. Primary focus lies on the gait parameters maximum peak load and step duration in relation to discharge date; however, secondary outcomes in other spatiotemporal gait parameters and additional validated clinical tests will be analyzed as well. This trial will contribute to existing knowledge of rehabilitation after hip fractures in the elderly, hopefully contributing to improved outcome for many affected.

## Trial status

This protocol (V1, 18/10/2016) started in March 2017 and the recruitment will approximately be completed in August 2018.

## Additional file

Additional file 1: SPIRIT checklist. (DOC 121 kb)

## Abbreviations

EMS: Elderly Mobility Scale
FAC: Functional Ambulation Categories
MMSE: Mini Mental State Examination
MUMC+: Maastricht University Medical Center+
RCT: Randomized controlled trial
UMCU: University Medical Center Utrecht
VAS: Visual Analogue Scale

## References

[1] Dhanwal D, Dennison E, Harvey N, Cooper C (2011). Epidemiology of hip fracture: Worldwide geographic variation. Indian J Orthop.

[2] Peeters CMM, Visser E, Van De Ree CLP, Gosens T, Den Oudsten BL, De Vries J (2016). Quality of life after hip fracture in the elderly: A systematic literature review. Injury.

[3] Beaupre LA, Binder EF, Cameron ID, Jones CA, Orwig D, Sherrington C (2013). Maximising functional recovery following hip fracture in frail seniors. Best Pract Res Clin Rheumatol.

[4] Vasarhelyi A, Baumert T, Fritsch C, Hopfenmüller W, Gradl G, Mittlmeier T (2006). Partial weight bearing after surgery for fractures of the lower extremity – is it achievable?. Gait Posture..

[5] Robling AG, Castillo AB, Turner CH (2006). Biomechanical and molecular regulation of bone remodeling. Annu Rev Biomed Eng.

[6] Klein-Nulend J, Bacabac RG, Mullender MG (2005). Mechanobiology of bone tissue. Pathol Biol.

[7] Hurkmans HLP, Bussmann JBJ, Benda E, Verhaar JAN, Stam HJ. Techniques for measuring weight bearing during standing and walking. Clin Biomech. 2003;18:576–89. 10.1016/s0268-0033(03)00116-5 12880705

[8] Hershko E, Tauber C, Carmeli E (2008). Biofeedback versus physiotherapy in patients with partial weight-bearing. Am J Orthop.

[9] Raaben M, Holtslag HR, Leenen LPH, Augustine R, Blokhuis TJ (2018). Real-time visual biofeedback during weight bearing improves therapy compliance in patients following lower extremity fractures. Gait Posture.

[10] Jiang Y, Norman KE (2006). Effects of visual and auditory cues on gait initiation in people with Parkinson’s disease. Clin Rehabil.

[11] Druzbicki M, Guzik A, Przysada G, Kwolek A, Brzozowska-Magoń A (2015). Efficacy of gait training using a treadmill with and without visual biofeedback in patients after stroke: A randomized study. J Rehabil Med.

[12] Baram Y, Lenger R (2012). Gait improvement in patients with cerebral palsy by visual and auditory feedback. Neuromodulation.

[13] Isakov E (2007). Gait rehabilitation: a new biofeedback device for monitoring and enhancing weight-bearing over the affected lower limb. Eura Medicophys.

[14] Hurkmans HL, Bussmann JB, Benda E, Verhaar JA, Stam HJ (2012). Effectiveness of audio feedback for partial weight-bearing in and outside the hospital: A randomized controlled trial. Arch Phys Med Rehabil.

[15] Raaben M, Holtslag H, Augustine R, van Merkerk R, Koopman B, Blokhuis T (2017). Technical aspects and validation of a new biofeedback system for measuring lower limb loading in the dynamic situation. Sensors.

[16] Tombaugh TN, McIntyre NJ (1992). The mini-mental state examination: a comprehensive review. J Am Geriatr Soc.

[17] Thabane L, Mbuagbaw L, Zhang S, Samaan Z, Marcucci M, Ye C (2013). A tutorial on sensitivity analyses in clinical trials: The what, why, when and how. BMC Med Res Methodol.

